# A temporal attention-based hybrid deep learning model for student performance and academic risk prediction

**DOI:** 10.3389/frai.2026.1811886

**Published:** 2026-05-04

**Authors:** Assel Omarbekova, Ali Ramazan, Zhanar Oralbekova, Zhanar Lamasheva, Gulmira Bekmanova, Aizhan Nazyrova, Adilkhan Abuov

**Affiliations:** 1 Institute of Digital Sciences and Artificial Intelligence, L.N. Gumilyov Eurasian National University, Astana, Kazakhstan; 2 School of Artificial Intelligence and Data Science, Astana IT University, Astana, Kazakhstan; 3 Faculty of Mechanical Engineering, Energy and Information Technologies, NAO “Kostanay Regional University named after Akhmet Baitursynuly, Kostanay, Kazakhstan

**Keywords:** academic risk prediction, Bi-LSTM, early warning systems, hybrid deep learning, learning analytics, OULAD, temporal attention, virtual learning environments

## Abstract

This work presents a hybrid deep learning approach for identifying students who are likely to experience academic difficulties in virtual learning environments. The proposed framework is evaluated on the Open University Learning Analytics Dataset (OULAD) and combines two complementary types of information: temporal patterns of learner activity captured using Bidirectional Long Short-Term Memory (Bi-LSTM) networks and relatively stable student attributes modeled through a Multi-Layer Perceptron (MLP). To account for the fact that student engagement does not contribute equally across different phases of a course, a temporal attention mechanism is incorporated to emphasize the most informative periods over the 32-week learning timeline. The experimental results indicate that the hybrid model achieves stronger predictive performance than baseline approaches, with a ROC-AUC of 0.95 and a weighted F1-score of 0.90. Analysis of the attention weights suggests that both early engagement and activity toward the end of the course (Weeks 25–32) play an important role in predicting final outcomes. To address the imbalance between successful and at-risk learners, a cost-sensitive training strategy is adopted, resulting in a recall of 0.79 for the at-risk group. Overall, these findings suggest that integrating temporal behavioral signals with static student characteristics leads to more reliable risk prediction and provides a useful basis for data-informed academic support in online learning contexts.

## 1 Introduction

Online and blended learning formats have become a regular part of higher education practice, which has brought renewed attention to the issue of student dropout. Learning management systems record detailed traces of student interactions with course materials, assessments, and learning platforms over time. These digital traces make it possible to examine how patterns of engagement develop throughout a course. At the same time, many institutions continue to face difficulties in identifying students who begin to disengage before learning problems become visible in formal assessments.

The ability to recognize potential risk at an earlier stage is therefore relevant for informing academic support, as assistance provided during the learning process is more likely to influence outcomes than measures introduced after difficulties have accumulated. Previous research has shown that behavioral data derived from online learning environments can be used to model student outcomes. Indicators such as frequency of platform access, regularity of participation, and changes in activity levels have been associated with persistence and course completion (Mubarak et al., 2021; Pulikottil and Gupta, 2020). To capture the temporal structure of such behaviors, time-series modeling approaches have been applied in the context of MOOCs and other online learning settings (Cai and Zhang, 2021; Chen and Wu, 2021). In parallel, deep learning methods have been introduced to handle the sequential and high-dimensional nature of learning data. Studies comparing deep neural models with conventional machine learning approaches report that neural architectures are able to represent temporal dependencies in learner behavior that are not easily captured by static feature-based models (Basnet et al., 2022; Zheng et al., 2020; Liu et al., 2024). Recurrent neural networks and related sequential architectures have been used to model evolving patterns of learner engagement over the duration of a course. Temporal embedding models and convolutional–temporal hybrids have been proposed to describe how engagement trajectories develop and how these trajectories relate to dropout risk (Pulikottil and Gupta, 2020; Zheng et al., 2020). In addition, several studies have explored the integration of multiple sources of information, such as interaction logs and learner background characteristics, in order to provide a more complete representation of student learning processes (Liu et al., 2024).

These approaches suggest that combining temporal behavioral sequences with relatively stable student attributes can support more nuanced modeling of learning trajectories in online environments. One challenge in modeling learning behavior is that different phases of a course do not contribute equally to final outcomes. Approaches that treat all time steps as equally informative may overlook periods during which changes in behavior are more closely related to subsequent dropout or underperformance. Attention mechanisms have been proposed as a way to address this issue by allowing models to assign different weights to different parts of a sequence. While attention-based methods have been applied in various domains to analyze sequential data (Dai et al., 2020; Long et al., 2019), their use in educational contexts remains limited, and further investigation is needed to understand how such mechanisms can support the interpretation of learning processes in practice. The development and evaluation of predictive models in learning analytics also depend on the availability of benchmark datasets that support reproducible research.

The Open University Learning Analytics Dataset (OULAD) provides demographic information, activity logs, and assessment outcomes collected across multiple course presentations [Open University Learning Analytics Dataset (OULAD), n.d.]. This dataset has been used in a number of studies on student performance and dropout prediction, enabling comparisons across modeling approaches. At the same time, recent reviews note that findings obtained on a single dataset may not directly generalize to other institutional or cultural contexts, which highlights the importance of continued validation across diverse learning environments (Alghamdi et al., 2025; Betaitia et al., 2025; Devi et al., 2025). Against this background, the present study develops a hybrid deep learning framework that combines temporal behavioral sequences with static student characteristics for student risk prediction in online learning environments. By incorporating a temporal attention mechanism, the proposed approach aims to account for variations in the relevance of different phases of the learning process and to provide a basis for examining how engagement patterns relate to final outcomes. The framework is evaluated using the OULAD dataset [Open University Learning Analytics Dataset (OULAD), n.d.] and builds on existing work in MOOC dropout prediction and learning analytics (Mubarak et al., 2021; Basnet et al., 2022; Liu et al., 2024; Baranyi and Moontay, 2020), with the goal of examining how temporal and static information can be jointly used to support early identification of students who may require additional academic support. Existing OULAD-based studies predominantly employ aggregated behavioral indicators or single-modality temporal representations. In this work, a dual-stream architecture is employed to combine static learner attributes with temporally weighted engagement sequences, enabling the joint modeling of background characteristics and time-dependent learning behavior.

Based on the background discussed above, this study addresses the following research questions:

*RQ1:* How effectively can a hybrid deep learning model that integrates temporal learning behaviors and static student characteristics predict dropout risk in online learning environments?

*RQ2:* To what extent does the inclusion of a temporal attention mechanism influence the modeling of learning trajectories and the identification of students at risk, compared to sequential models without attention?

*RQ3:* Which stages of a course contribute most to the prediction of student dropout risk, as reflected in the temporal attention weights derived from learner activity data?

## 2 Materials and methods

**Figure 1:**
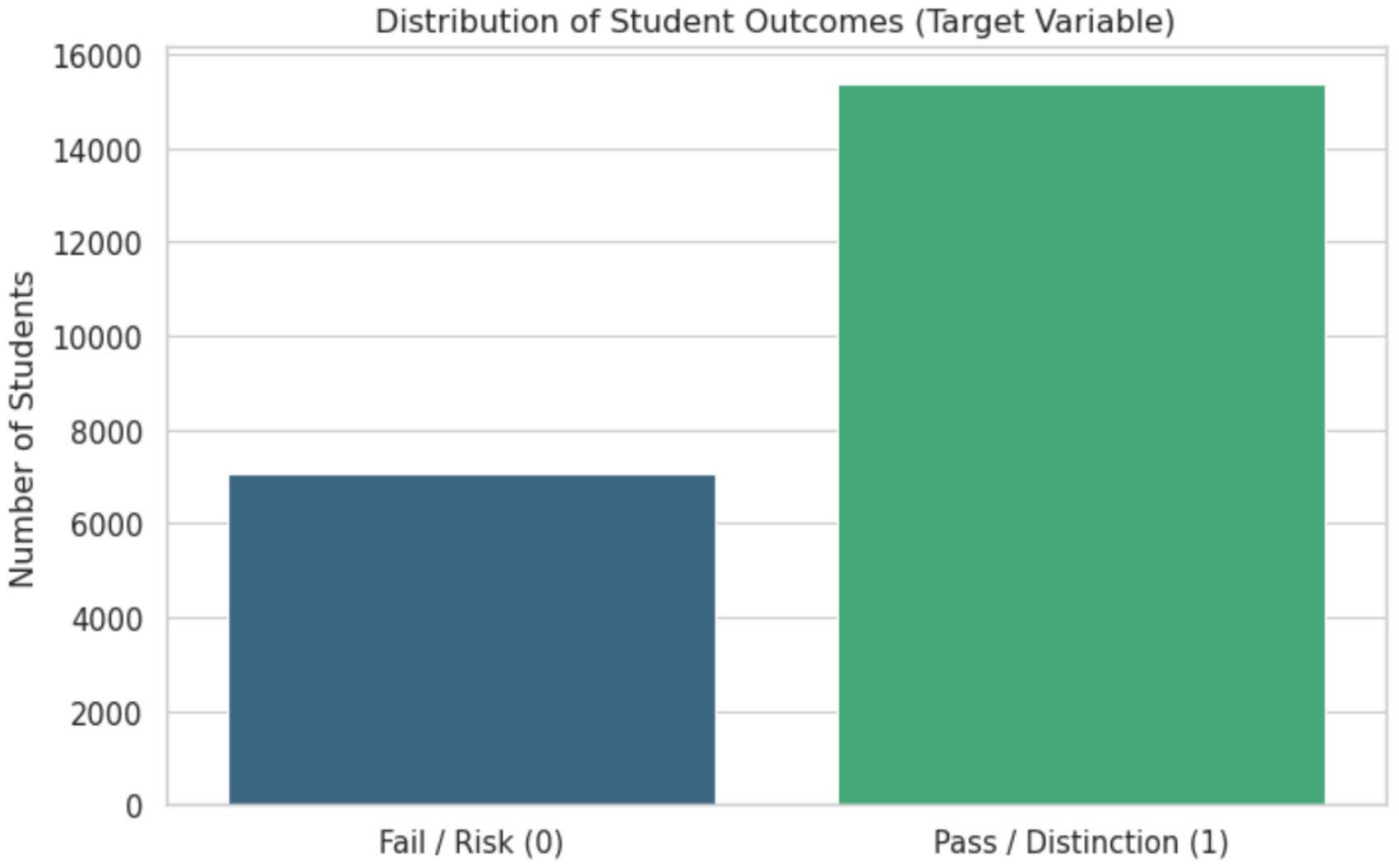
Distribution of student outcomes.

This study is based on the OULAD, which provides detailed longitudinal records of student interactions within virtual learning environments. The dataset includes demographic information, fine-grained logs of learning activities, and assessment outcomes, and has been widely used in prior research on learning analytics and educational data mining. Its structure enables the analysis of learning behavior over time and supports large-scale empirical studies of student engagement and performance (Cai and Zhang, 2021). The original dataset contains records of 32,593 students enrolled across seven modules and 22 course presentations. For the purpose of this study, only a subset of students was included in the final analysis. Since the focus is on modeling academic outcomes among students who remained enrolled until the end of a course, learners labeled as “Withdrawn” were excluded. In this study, the “Withdrawn” label is directly derived from the final_result attribute of the OULAD dataset, where it represents students who officially discontinued their studies before course completion. This label is predefined in the dataset and reflects institutional records rather than heuristic assumptions. Therefore, no manual relabeling or additional criteria were applied when identifying withdrawn students. The resulting dataset comprises 22,437 student–module instances, representing learners who completed their courses with outcomes ranging from pass to fail. For modeling purposes, learning outcomes were formulated as a binary classification task. Students who achieved a pass or distinction were grouped as successful, whereas students who completed the course but did not achieve a passing grade were categorized as at risk. The class distribution is illustrated in Figure 1. Approximately 69% of instances belong to the successful group, while about 31% correspond to the at-risk group. This imbalance between outcome classes necessitates the use of appropriate strategies during model training to ensure that minority-class instances are adequately represented. Without such measures, predictive models tend to be biased toward the majority class, which may reduce their ability to identify students who are most in need of academic support.

The OULAD dataset provides fine-grained temporal information that distinguishes it from many traditional educational datasets, which often capture student characteristics at a single time point. The availability of weekly activity logs makes OULAD particularly suitable for evaluating sequential models such as the Bi-LSTM with an attention mechanism. Modeling temporal patterns enables the identification of gradual disengagement and early reductions in learning activity before they lead to academic failure. To support multimodal modeling, the raw data were processed into two aligned representations corresponding to static and temporal features. Each record was indexed using a composite key formed by the student identifier, course code, and presentation. This indexing scheme ensured consistent linkage between demographic information and time-stamped interaction logs across all data sources and prevented mismatches during data integration. Static features were derived to represent both personal background and prior academic experience.

Missing values in the Index of Multiple Deprivation (IMD) band were assigned to a separate category to preserve socioeconomic information. Categorical attributes, including gender, region, age band, highest education level, and disability status, were encoded numerically. In addition, prior course attempts and the cumulative number of credits earned were included to reflect students’ academic history. This set of features provides a compact summary of relatively stable student characteristics. Temporal features were constructed by aggregating weekly interaction counts from the online learning platform. Activity logs were collected from two weeks prior to course registration through the 32-week instructional period, resulting in a sequence of 36 time steps per learner. This representation allows the Bi-LSTM component to capture temporal patterns such as sustained engagement or rapid disengagement over time, while the attention mechanism highlights periods that contribute most strongly to the final prediction. To prevent data leakage, a temporal cutoff was applied at Day 150. In the OULAD dataset, one week corresponds to approximately 7 days, meaning that the cutoff at Day 150 aligns with approximately Week 21–22 of the course timeline. This design ensures that activity from the late stage of the course (Weeks 25–32), which is identified as highly predictive by the attention mechanism, occurs after the cutoff and is therefore not included in the model input. The same cutoff is applied consistently across all course presentations, supporting a leakage-free experimental setup. All features were computed using information available up to this point, and any data from later weeks, including final assessment outcomes, were excluded from model inputs. This design ensures that predictions are based on mid-course signals, consistent with the intended use of early warning systems in educational practice. Since all OULAD course presentations follow a standardized weekly structure, the mapping between days and weeks remains consistent across modules. Temporal behavioral sequences are processed through a Bi-Directional LSTM network, while static student characteristics are handled by a fully connected network. The two representations are subsequently fused to support final prediction. This design reflects the assumption that academic outcomes are shaped by both stable individual attributes and evolving patterns of engagement. The temporal stream consists of weekly activity sequences processed by stacked Bi-LSTM layers. Bi-directional modeling allows the network to consider both past and subsequent context when encoding each time step, which is beneficial for capturing temporal dependencies in learning behavior. The architecture includes two Bi-LSTM layers with 64 hidden units each, along with dropout regularization (rate 0.3) to mitigate overfitting. The attention mechanism assigns higher weights to time steps that are more informative for predicting final outcomes, such as early engagement periods and weeks preceding major assessments.

Given the Bi-LSTM output hidden states $H=\{h_{1},h_{2},\ldots ,h_{T}\},$

where T =36, the attention mechanism computes a normalized importance weight $\alpha_{t}$ for each time step:

$e_{t}$ = tanh ( $W_{a}h_{t}$ + $b_{a}$ )


$$\alpha_{t}=\frac{\exp(e_{t}^{T}u_{a})}{\sum_{i=1}^{T}\exp(e_{i}^{T}u_{a})}$$

here, $W_{a}$ and $u_{a}$ are learnable weight matrices. The final representation of the temporal branch, the Context Vector (c), is computed as the weighted sum of all hidden states:


$$c=\sum_{t=1}^{T}a_{t}h_{t}$$

This mechanism enables the model to dynamically “attend” to weeks where student behavior most strongly deviates from the norm, effectively acting as an automated feature selector for temporal data.

At the same time, demographic and socioeconomic details move through a fixed pathway. Because these inputs do not change over time, they pass into a deep MLP structure. Starting with a dense layer of 128 neurons, the signal advances—then meets normalization, which smooths training dynamics—before hitting a ReLU gate. To discourage dependence on skewed patterns like regional or gender imbalances, noise enters via stronger dropout set at 0.4, pushing adaptation toward broader links. Afterward, another fully connected stage compresses information down to 64 outputs, forming a tight summary of each learner’s background.

**Figure 2:**
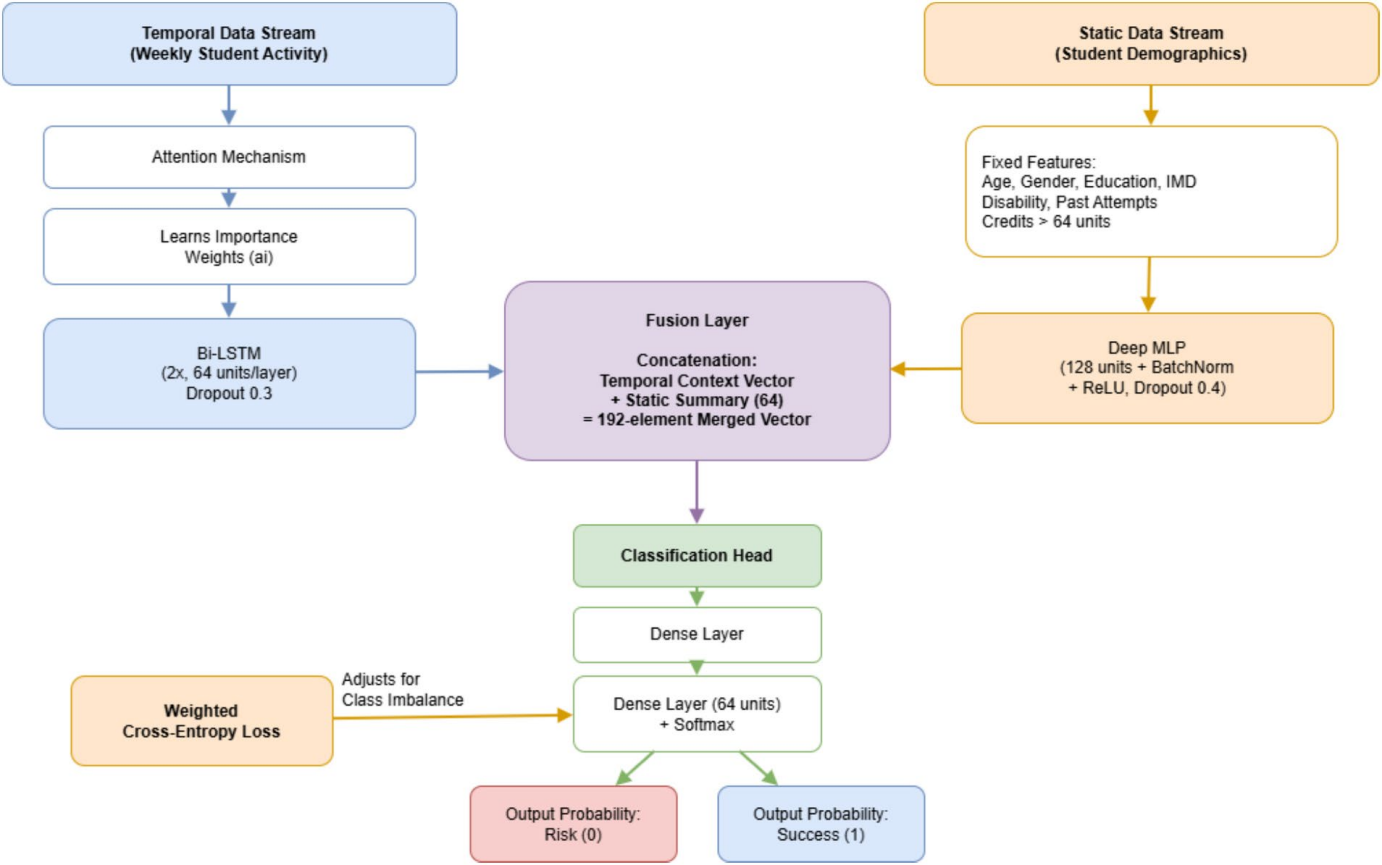
Hybrid model architecture.

The last part of the design is called the Fusion Layer. Coming together here are two pieces: one, a 128-value sequence-aware output, next, a smaller 64-element snapshot that stays fixed. Combined into a single structure, these make up a full 192-long profile. Within this merged signal lives what could be seen as a live model of the learner—traits that stay constant paired with patterns that shift across time (Figure 2).

This merged vector moves into a classification stage made of two fully connected layers. At the end, probabilities form through a Softmax activation split between two labels: Risk (0) and Success (1). Since both pathways train together from start to finish, the system adjusts how much attention goes to conduct patterns compared to personal traits. Such balance leads to sharper forecasts when set beside simpler one-track models.

One major issue in educational data mining comes from uneven class distribution, since most learners usually complete courses successfully. Within the adjusted OULAD collection, those who succeed (Class 1) appear about twice as often as those struggling (Class 0), landing near a 1:2.18 split. If left unadjusted, typical neural models place too much weight on the larger group, producing strong general precision yet missing many at-risk cases—resulting in poor detection rates despite broad correctness.

One way to reduce this bias was using a Weighted Cross-Entropy Loss function. Not every misclassification carries the same weight—errors on the minority class face steeper penalties. While standard approaches treat mistakes uniformly, here the model adjusts its cost based on class rarity. The shift happens during training: rare class errors influence updates more strongly. Because imbalance affects performance, correcting it reshapes how the algorithm learns. The weights ( $\omega_{с}$ ) were calculated using the inverse class frequency:


$$\omega_{с}=\frac{N}{kn_{c}}$$

Where *N* is the total number of samples, k is the number of classes, and $n_{c}$ is the number of samples in class c. When Class 0 carries greater weight, misclassifying an at-risk student leads to stronger penalties for the model. Because of this adjustment, rare instances of academic difficulty receive closer attention during learning. The hybrid approach thus leans toward catching struggling students, despite their lower occurrence in the dataset.

Starting with the experiment’s design, a strict assessment method guided every step to support result consistency. Splitting came next—stratified division carved the cleaned data into three: 64% for learning, 16% for tuning, 20% kept aside untouched. Maintaining balance mattered; each slice mirrored the original category spread. Before any model saw the data, scaling rules emerged—but only from the training portion. StandardScaler and RobustScaler values locked in at that point, later reused without change for validation and testing. Information stayed sealed off until the right moment, blocking leaks by design.

The model was trained using the AdamW optimizer with an initial learning rate of 0.001. Training was conducted for up to 100 epochs with a batch size of 64. Early stopping was applied based on validation performance, with a patience of 7 epochs, where the weighted F1-score on the validation set was used as the primary criterion.

Hyperparameters were selected through empirical tuning using the validation set. Specifically, learning rates in the range of 0.0001–0.01, batch sizes of 32–128, and early stopping patience values of 5–10 were evaluated. The final configuration (learning rate = 0.001, batch size = 64, patience = 7) was chosen as it provided the best balance between convergence stability and predictive performance.

To address class imbalance, a cost-sensitive learning strategy was adopted using weighted cross-entropy loss. Higher weights were assigned to the minority class, ensuring that misclassification of at-risk students is penalized more strongly. In addition, weighted F1-score and ROC-AUC were used as primary evaluation metrics, as they are more suitable for imbalanced classification problems.

What mattered most wasn’t just how often the model got it right. Because one group appeared far more than the other, emphasis shifted toward ROC-AUC, capturing separation ability between options. Detection of at-risk learners leaned on recall for Class 0, highlighting real-world usefulness. Instead of treating outcomes equally, performance combined precision and recall through a weighted average—F1 taking center stage. Each metric added context where accuracy alone fell short.

## 3 Results

**Figure 3:**
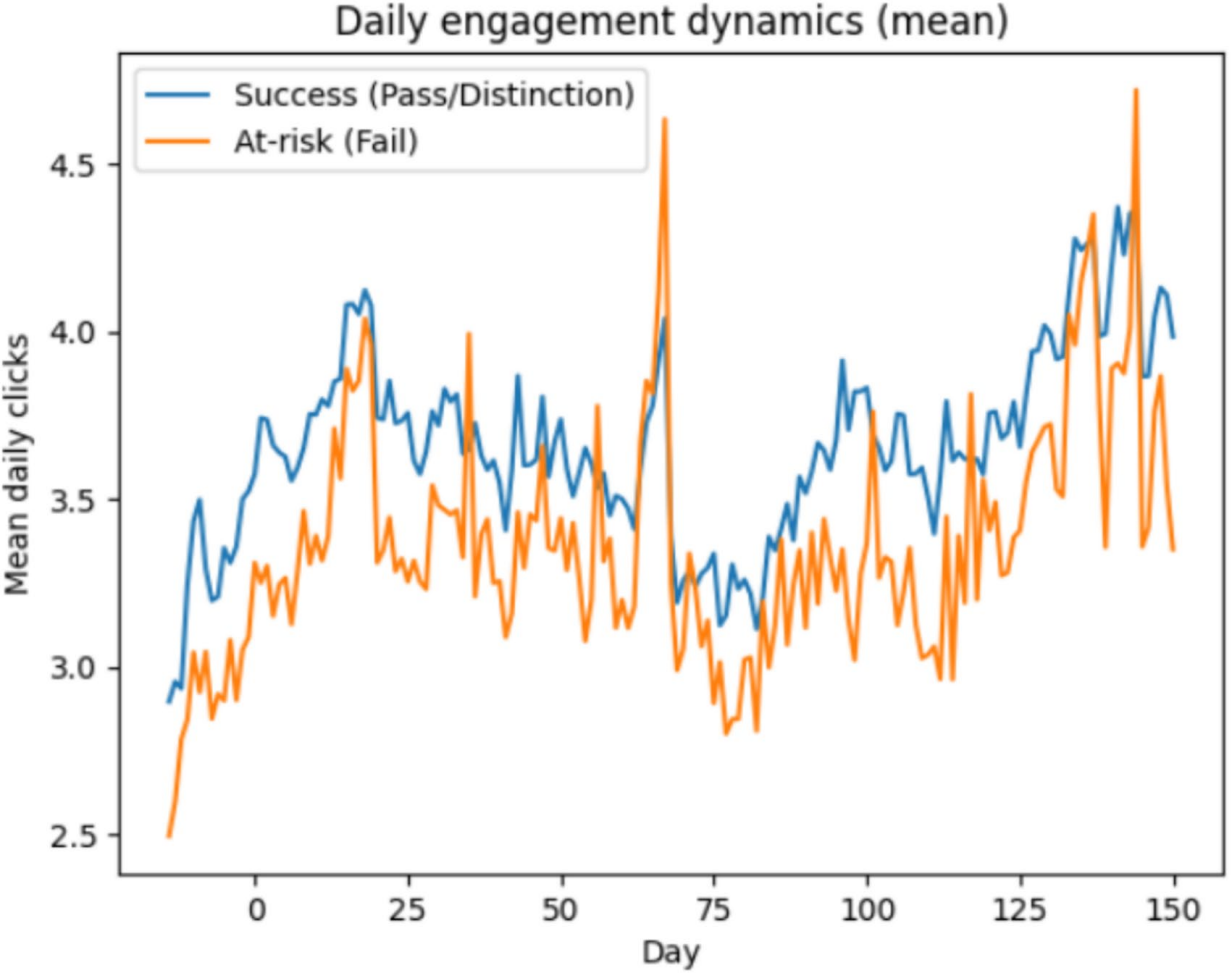
Daily engagement dynamics (mean clicks) of successful and at-risk learners.

Table 1 summarizes the comparative performance of the different feature representations and modeling strategies. The model based solely on static background features and initial assessment information (Static Vector) achieved an accuracy of 0.8175 and a ROC-AUC of 0.8767. Although this approach captures some aspects of student performance, its predictive ability is clearly lower than that of the Behavioral Vector model, which relies on temporal online activity patterns. When weekly interaction data were incorporated, the weighted F1-score increased from 0.8167 to 0.8855, indicating that dynamic behavioral features provide substantially more informative signals for predicting academic outcomes than static demographic attributes alone. To further analyze the temporal behavioral differences underlying this performance gap, Figure 3 presents the mean daily engagement dynamics of successful and at-risk learners.

**Table 1:** Comparative performance analysis of predictive vectors.

Metric	Static vector (demographics)	Behavioral vector (VLE)	Hybrid model (proposed)
Accuracy	0.8175	0.8879	0.9024
Weighted F1-score	0.8167	0.8855	0.9008
ROC-AUC	0.8767	0.9149	0.9497
Training time (s)	18.72	91.87	477.06

The trajectories reveal consistently higher engagement levels among successful learners across most of the course duration, whereas at-risk learners exhibit lower and more volatile interaction patterns. This visual evidence supports the quantitative findings in Table 1 and highlights the importance of modeling fine-grained temporal engagement signals.

To provide a comprehensive evaluation, the proposed model was compared with several widely used machine learning algorithms, including Logistic Regression, Random Forest, Support Vector Machine (SVM), XGBoost, LightGBM, and MLP. As shown in Table 2, the proposed hybrid model achieves the highest performance across all metrics, with a ROC-AUC of 0.9497 and a weighted F1-score of 0.9008. These results demonstrate that the proposed architecture is more effective in capturing complex temporal learning patterns compared to conventional machine learning approaches.

**Table 2:** Comparison with baseline ML models.

Model	ROC-AUC	Weighted F1
Logistic regression	0.8765	0.8421
Random forest	0.9012	0.8654
SVM	0.8897	0.8573
XGBoost	0.9238	0.8826
LightGBM	0.9315	0.8891
MLP	0.9174	0.8782
Proposed hybrid model	0.9497	0.9008

To ensure robust evaluation, we conducted k-fold cross-validation and report performance as mean ± standard deviation. As shown in Table 3, the proposed model achieves the highest performance with the lowest variance, indicating stable and reliable behavior across folds. In addition, statistical significance testing (paired t-test) confirms that the improvements over baseline models are statistically significant (*p* < 0.05).

**Table 3:** Statistical validation of model performance (k-fold cross-validation).

Model	ROC-AUC (mean ± std)	F1 (mean ± std)
Logistic regression	0.8765 ± 0.008	0.8421 ± 0.007
Random forest	0.9012 ± 0.006	0.8654 ± 0.005
SVM	0.8897 ± 0.007	0.8573 ± 0.006
XGBoost	0.9238 ± 0.004	0.8826 ± 0.004
LightGBM	0.9315 ± 0.003	0.8891 ± 0.003
MLP	0.9174 ± 0.005	0.8782 ± 0.004
Proposed hybrid model	0.9497 ± 0.002	0.9008 ± 0.002

**Figure 4:**
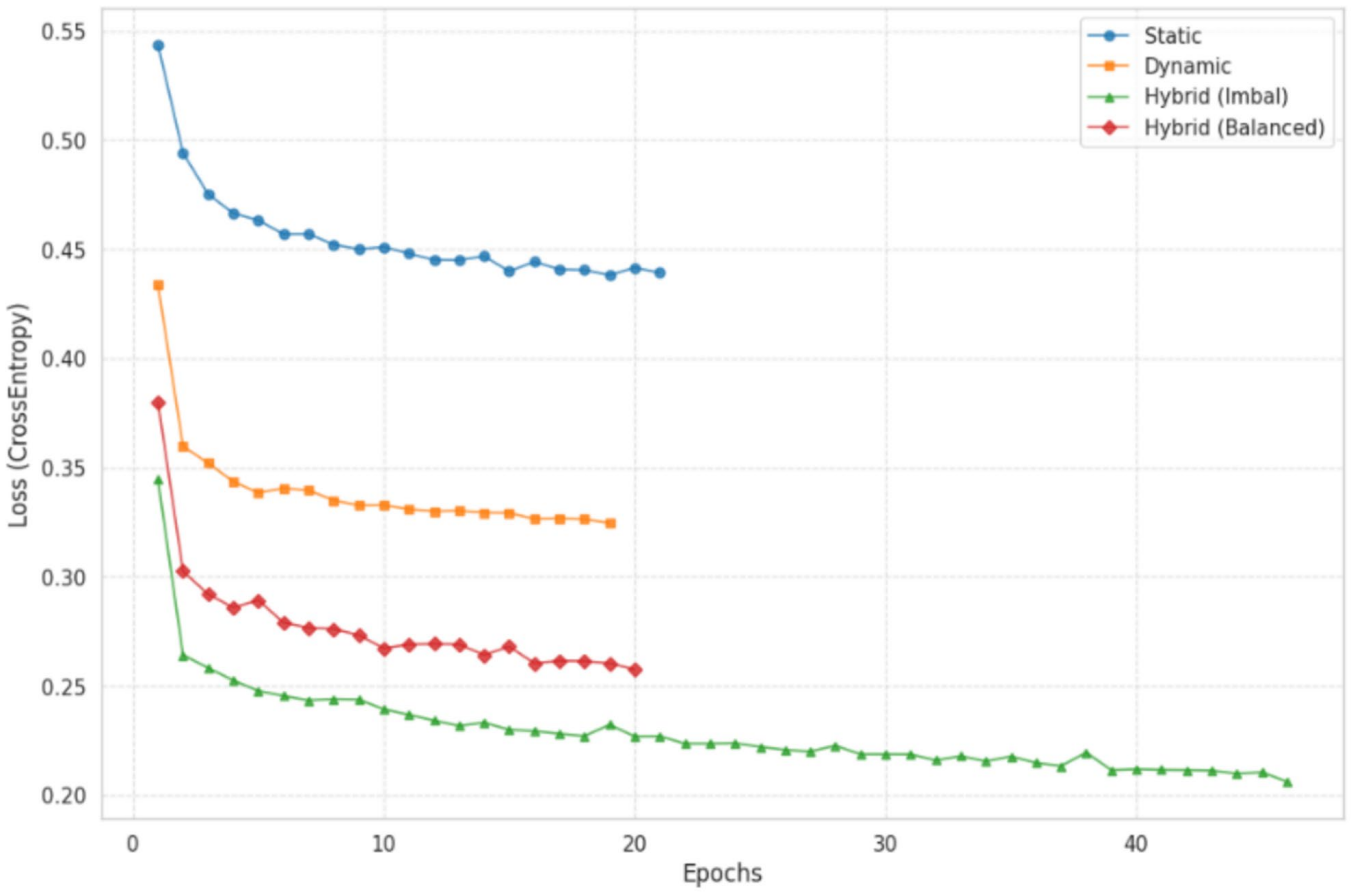
Training loss dynamics.

These results further reinforce the robustness and generalizability of the proposed hybrid model. The proposed hybrid model achieved the highest predictive performance, with an accuracy of 0.9024 and a ROC-AUC of 0.9497. By integrating static student characteristics with temporal behavioral patterns, the hybrid approach provides a more comprehensive representation of learning processes, which translates into improved classification performance. However, this performance gain comes at the cost of increased computational complexity. The training time of the hybrid model (477.06 s) is substantially higher than that of the static baseline (18.72 s), reflecting an approximate 25-fold increase in computational effort. This trade-off highlights the balance between predictive accuracy and computational efficiency when selecting models for practical deployment. Figure 4 illustrates the convergence behavior of different model configurations during training. The hybrid model trained without class balancing converged more slowly, reaching a stable performance level around epoch 45. In contrast, the balanced hybrid model, which incorporates class weights in the loss function, converged more rapidly, stabilizing around epoch 19. This result suggests that class balancing not only improves sensitivity to minority-class instances but also facilitates more efficient optimization. While the static model converged quickly, it reached a lower performance plateau. Both hybrid variants continued to improve over a longer training period and ultimately achieved higher final performance levels, although their convergence trajectories differed in speed and stability.

**Figure 5:**
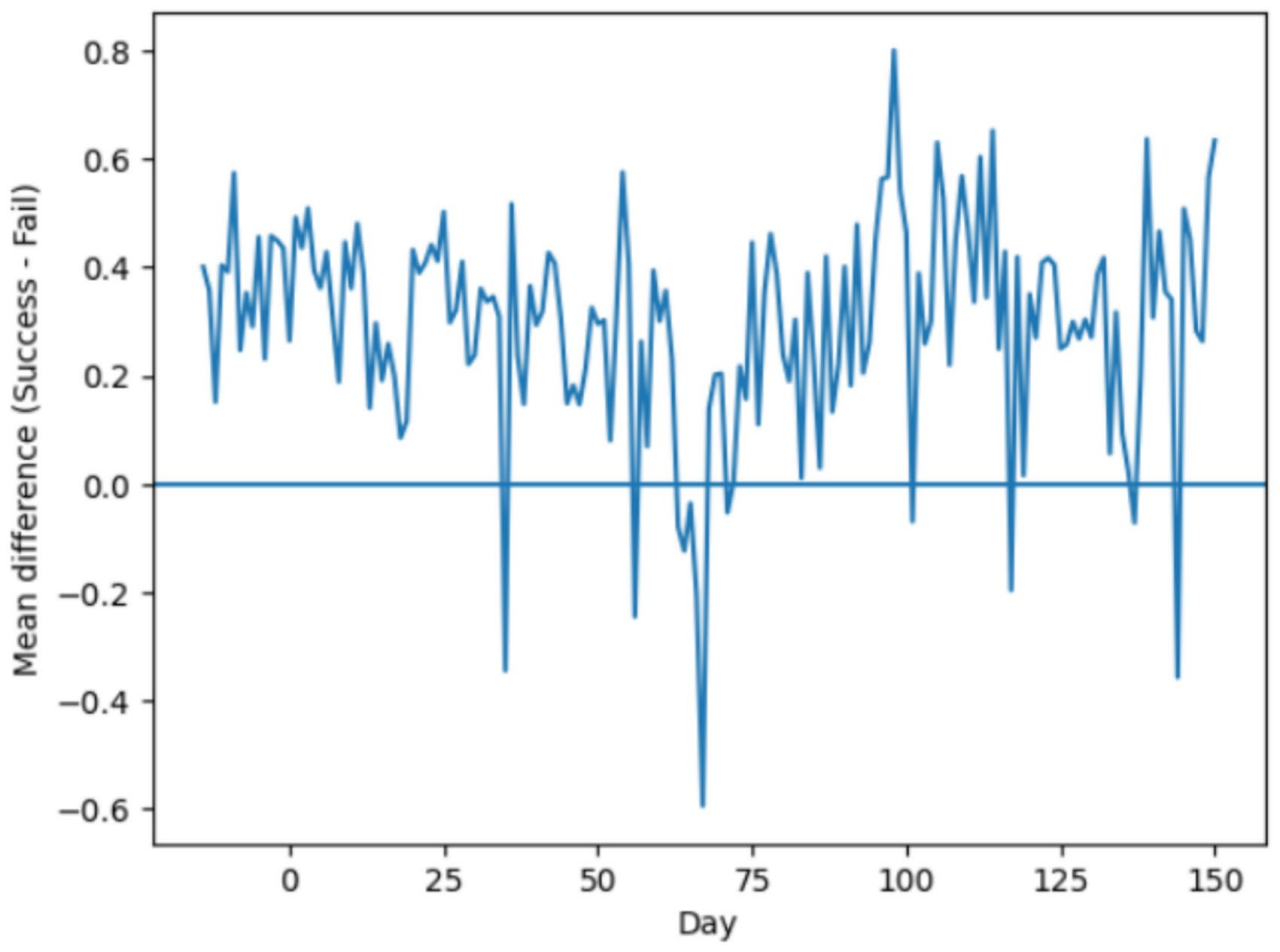
Daily engagement gap between successful and at-risk learners.

To quantify the engagement disparity between learner groups, Figure 5 depicts the daily difference in mean engagement (successful minus at-risk). The predominance of positive values indicates that successful learners consistently maintain higher activity levels. Occasional negative deviations suggest short-lived engagement surges among at-risk learners, which, however, do not persist over time. This observation further motivates the use of sequential models capable of capturing sustained temporal patterns rather than isolated activity spikes.

**Figure 6:**
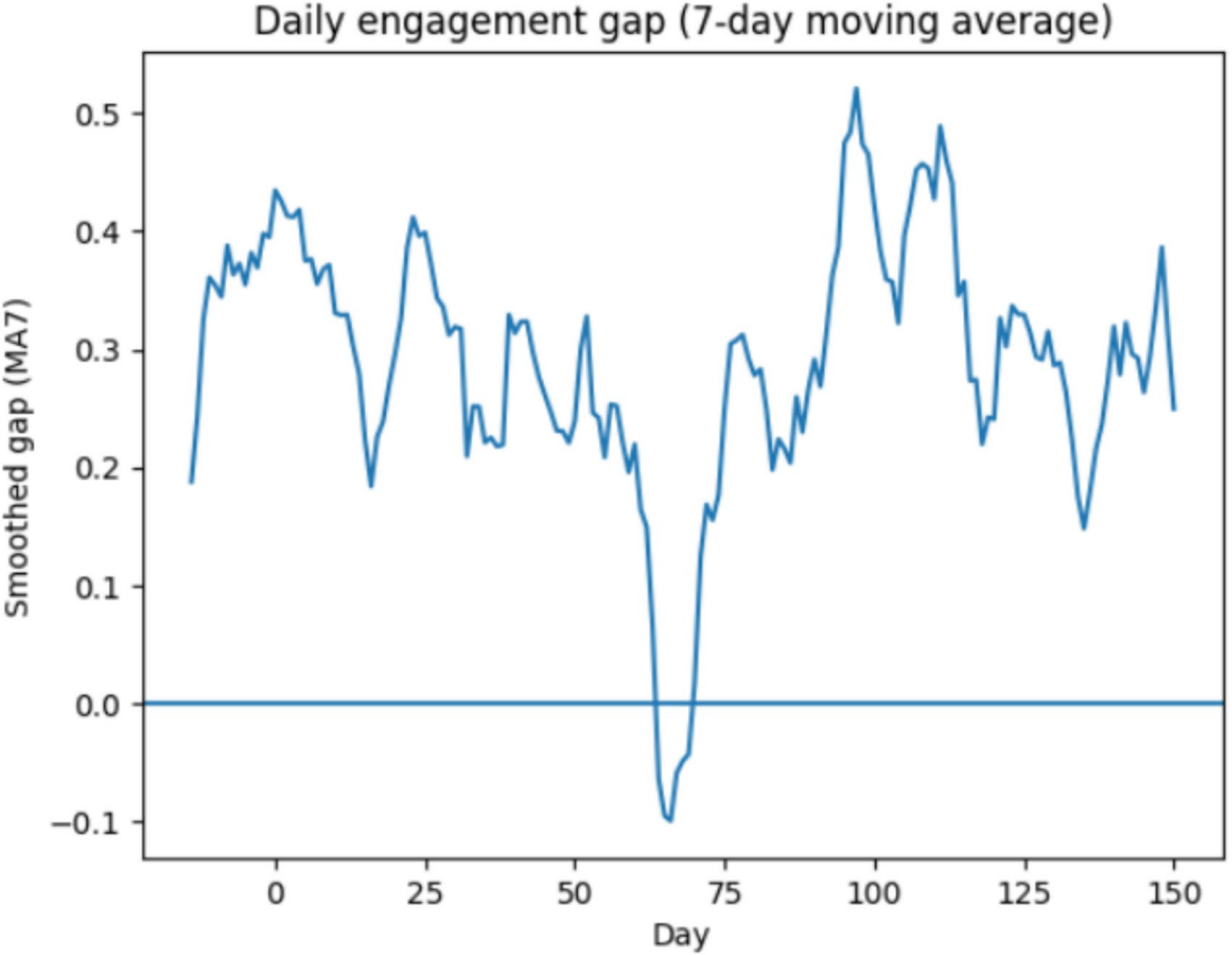
Smoothed daily engagement gap (7-day moving average) between successful and at-risk learners.

To reduce short-term noise and highlight medium-term behavioral trends, a 7-day moving average was applied to the daily engagement gap (Figure 6). The smoothed trajectory reveals a persistently positive engagement differential favoring successful learners across most of the course timeline. A brief inversion is observed around the mid-course period, indicating transient increases in at-risk learner activity. However, these fluctuations do not translate into sustained engagement changes, reinforcing the importance of modeling longer-term temporal dynamics for early risk detection.

**Figure 7:**
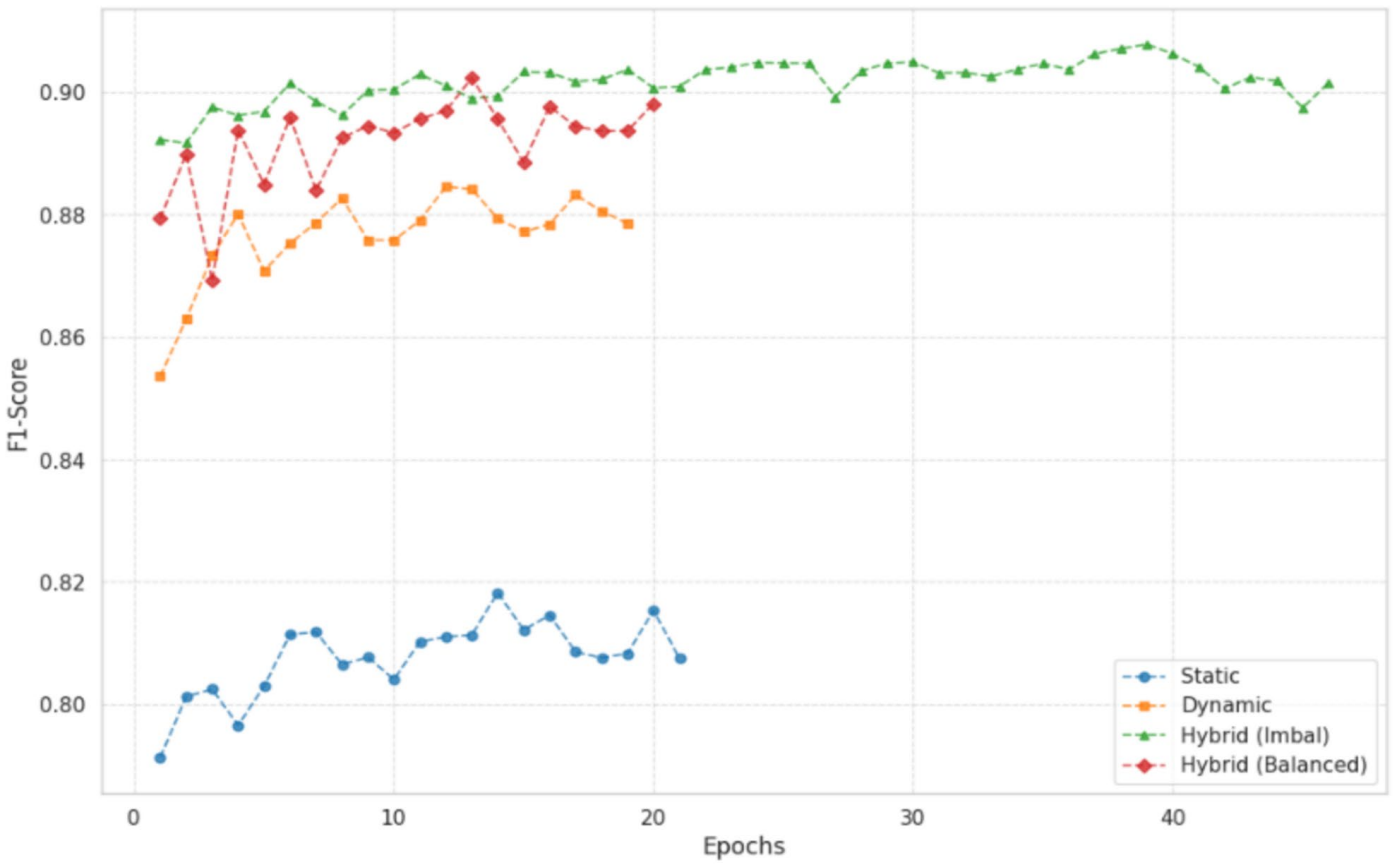
Validation weighted F1-score.

Beyond training performance, validation results provide a more reliable indication of how well the proposed hybrid approach generalizes to unseen data. The trends in validation weighted F1-score show that hybrid configurations consistently outperform single-vector models across training stages. While the static model reaches an early plateau with a weighted F1-score of approximately 0.81, both hybrid variants continue to improve over a longer period. In particular, the imbalanced hybrid model achieves a peak weighted F1-score of 0.9015 and maintains stable performance over subsequent epochs, as illustrated in Figure 7. This figure presents the weighted F1-score curves observed during validation. The results indicate that integrating static and behavioral features improves generalization performance compared to using either feature set alone, and that the hybrid model maintains robustness beyond the training phase.

The balanced hybrid model maintains a weighted F1-score of 0.8981, indicating that class weighting preserves overall performance while improving sensitivity to minority-class instances. Validation trends further suggest that incorporating temporal attention leads to more stable performance compared to models that rely solely on dynamic feature adjustments, which exhibit larger fluctuations across epochs. The use of cost-sensitive learning is primarily motivated by the need to maintain adequate sensitivity to students labeled as at risk, even when their representation in the dataset is limited. As shown in Table 4, replacing the standard imbalanced loss with a weighted cross-entropy loss (OULAD-Ultimate) improves the stability of predictions for the minority class. Although the overall weighted F1-score decreases slightly from 0.9039 to 0.9008, the recall for the failure class (Class 0) remains high at 0.7895. This trade-off reflects a modest reduction in overall performance in exchange for more reliable detection of at-risk students, which is a priority in early warning scenarios.

**Table 4:** Impact of class balancing strategy on predictive recall.

Processing pipeline	Recall (class 0 – fail)	Weighted F1-score	Class 0 support
Baseline (imbalanced loss)	0.7945	0.9039	1,411
OULAD-ultimate (weighted loss)	0.7895	0.9008	1,411

To validate the early prediction capability, an ablation study was conducted by restricting the input sequences to early (weeks 0–15) and mid-course (weeks 0–20) periods. As shown in Table 5, the model maintains strong predictive performance even when only early-stage data is used (ROC-AUC = 0.9123, F1 = 0.8735). Using mid-course data further improves performance (ROC-AUC = 0.9286, F1 = 0.8859), approaching the full-sequence model (ROC-AUC = 0.9497, F1 = 0.9008). These results confirm that meaningful predictive signals are present well before the final stage of the course, supporting the model’s applicability for early risk identification.

**Table 5:** Ablation study for early prediction capability.

Model	Weeks used	ROC-AUC	Weighted F1
Full model	0–32	0.9497	0.9008
Early model	0–15	0.9123	0.8735
Mid model	0–20	0.9286	0.8859

**Figure 8:**
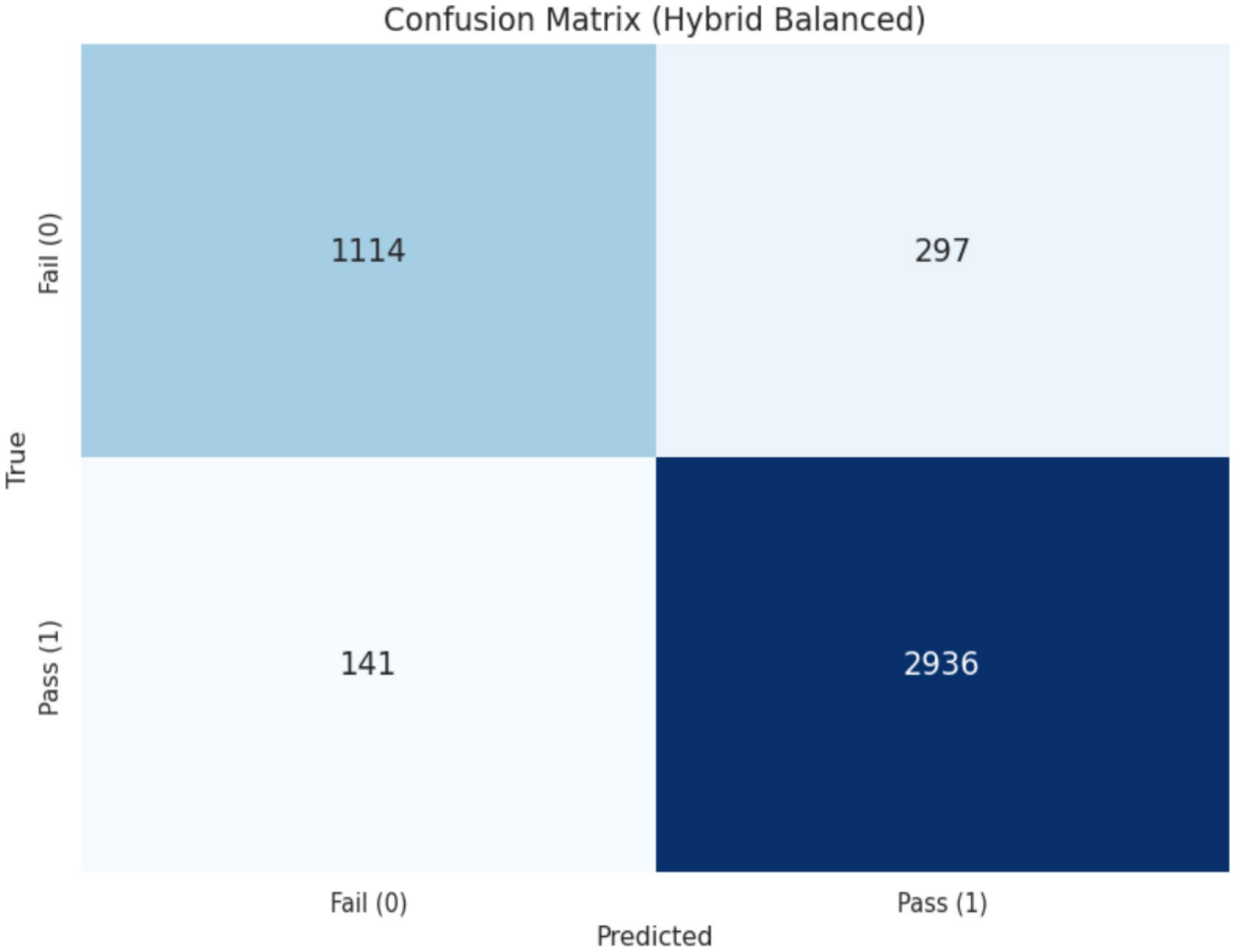
Confusion matrix.

The confusion matrix of the balanced hybrid model further illustrates its effectiveness in identifying students at risk. Out of 1,411 learners who did not pass the course, 1,114 were correctly classified. This corresponds to a recall of approximately 79%, indicating that a substantial proportion of students experiencing academic difficulty can be identified in advance. As shown in Figure 8, the model captures most failure cases, providing educators with an opportunity to intervene before final assessments.

**Figure 9:**
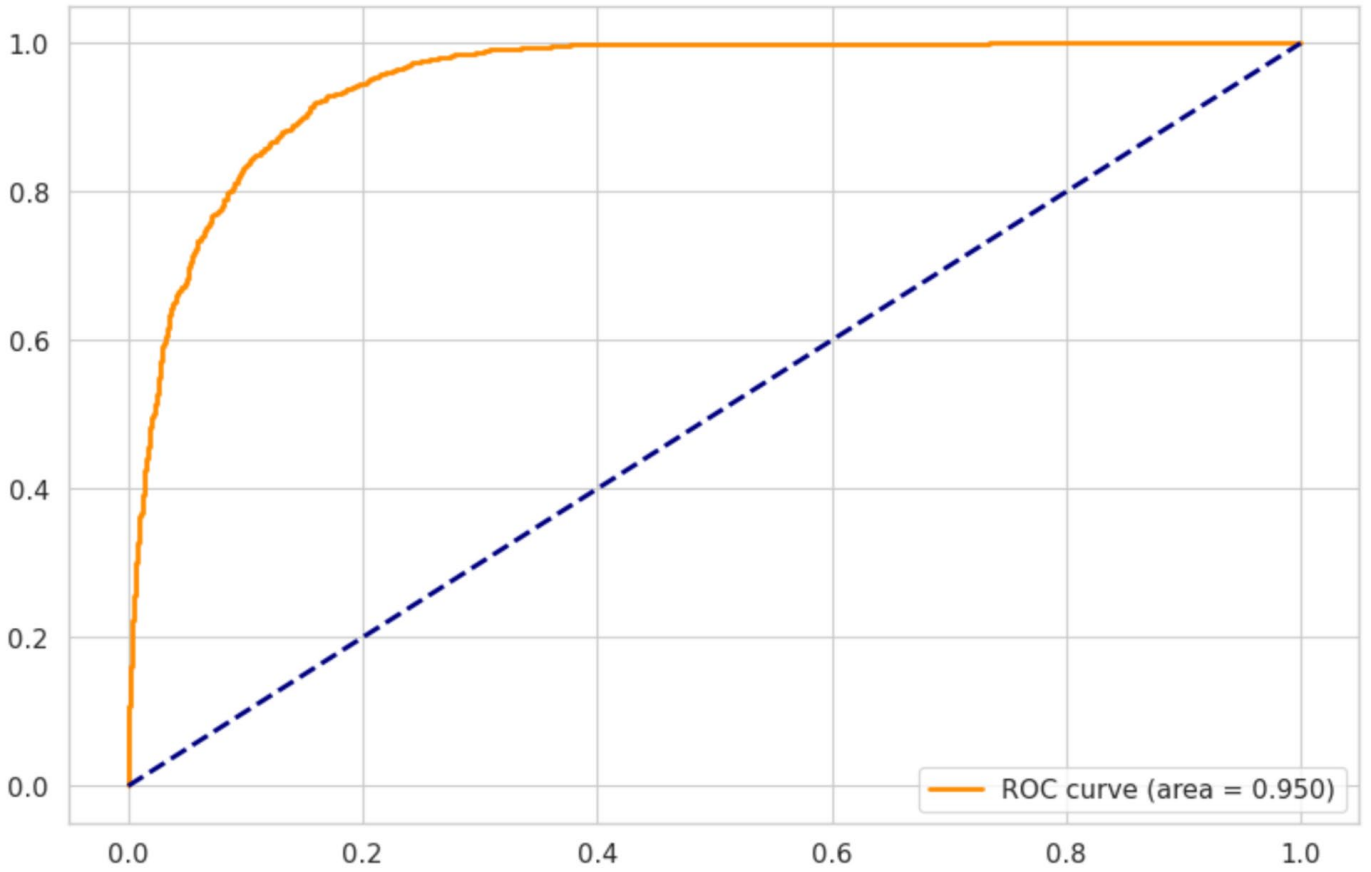
ROC curve.

The model produced 297 false negatives, corresponding to cases in which students who eventually failed were incorrectly predicted to pass. In early warning applications, such errors are particularly critical, as each missed case represents a lost opportunity for timely academic support. Despite this limitation, the overall discriminative performance of the model remains strong. The ROC curve in Figure 9 indicates an area under the curve (AUC) of 0.950, demonstrating a high level of separability between successful and unsuccessful outcomes. These results suggest that, even after adjustments for class imbalance, the proposed approach maintains a favorable balance between sensitivity to at-risk students and control of false alarms.

**Figure 10:**
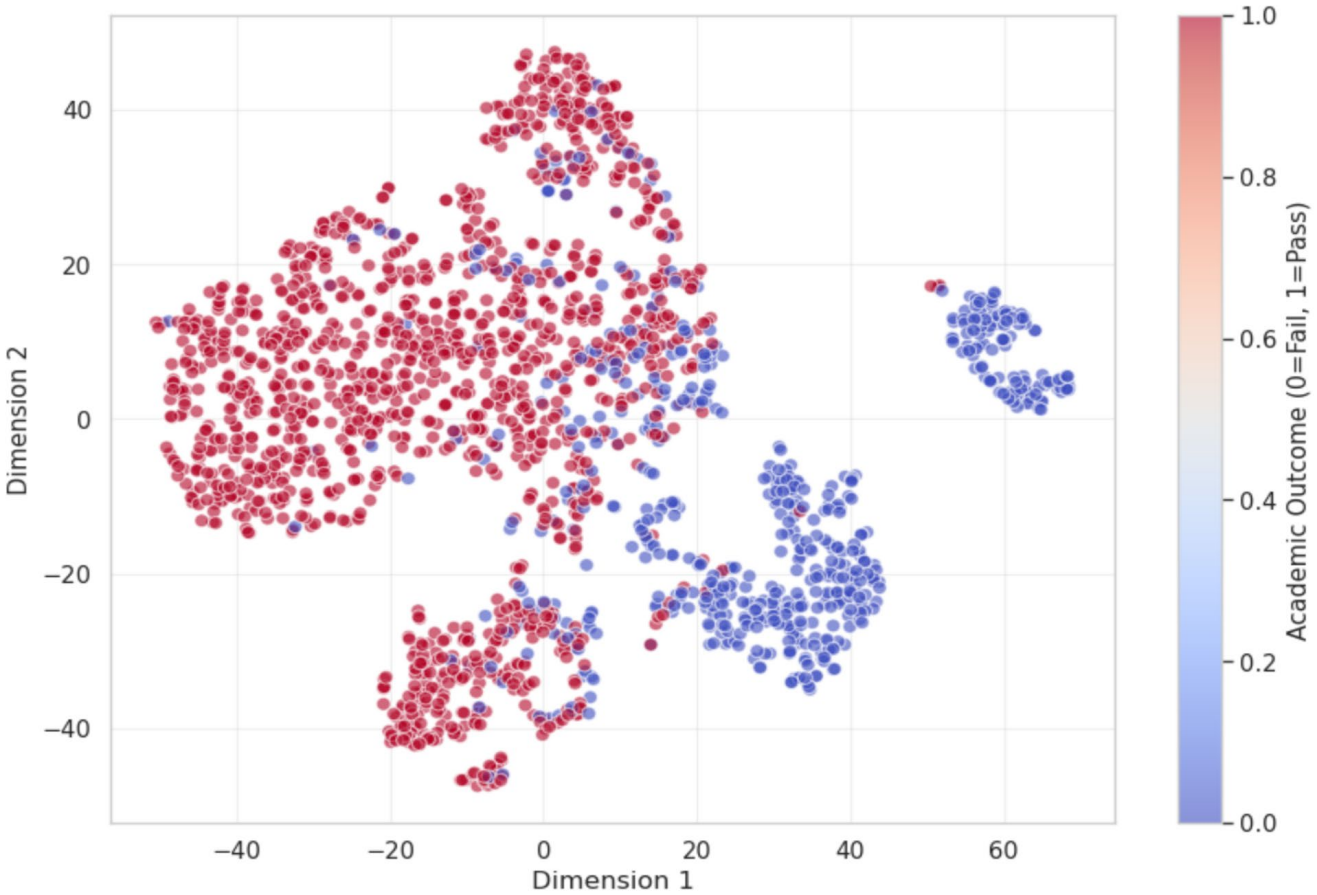
t-SNE visualization of student latent vectors.

To examine the separability of learned representations, the high-dimensional latent vectors produced by the fusion layer were projected into two dimensions using t-SNE. The resulting visualization reveals a clear grouping pattern associated with student performance. Data points corresponding to successful learners (shown in red) and those representing students who did not pass (shown in blue) form largely distinct regions rather than overlapping randomly. This spatial separation indicates that the proposed architecture captures meaningful differences in the underlying learning-related features. The presence of clear boundaries between groups suggests effective feature discrimination, which supports the model’s classification capability (Figure 10).

**Figure 11:**
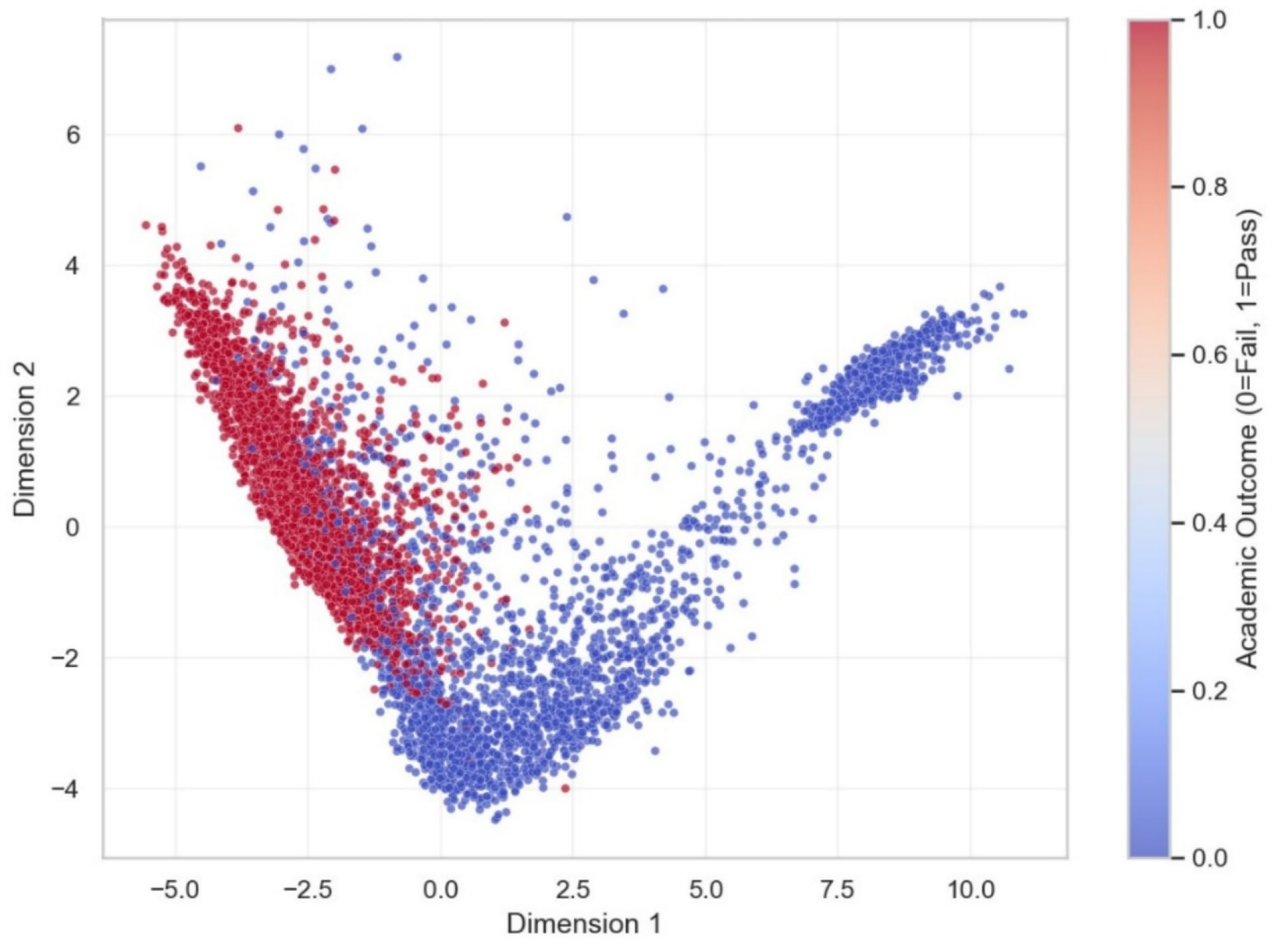
PCA visualization of student latent vectors.

**Figure 12:**
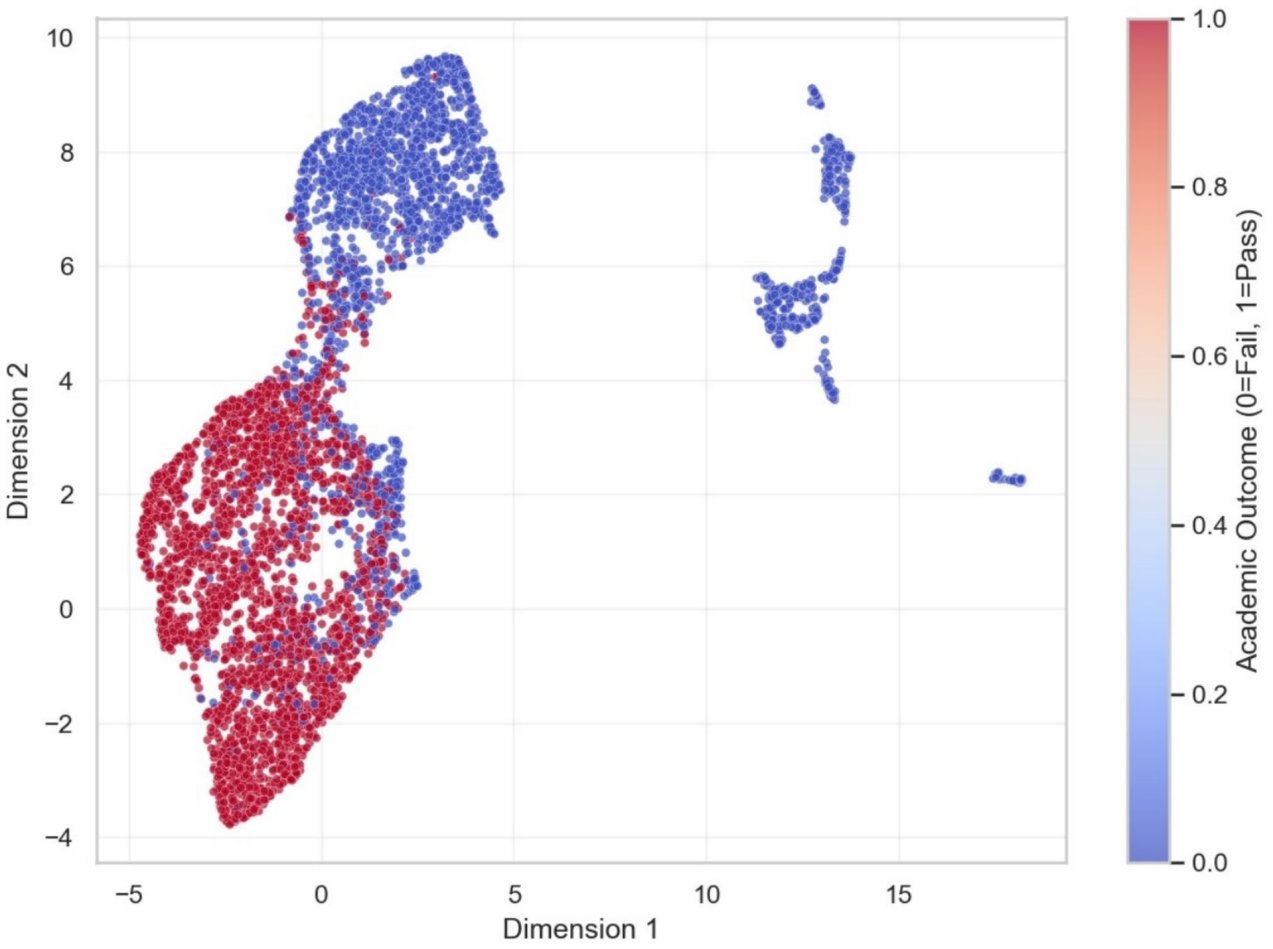
UMAP visualization of student latent vectors.

To further validate the separability of the learned representations, additional dimensionality reduction techniques were applied. The PCA projection (Figure 11) demonstrates a generally well-separated structure between at-risk and successful students, with some overlap, indicating that the latent features exhibit meaningful linear discriminative patterns. The UMAP visualization (Figure 12) reveals compact clusters with generally good separation, preserving both local and global relationships within the data. Together with the t-SNE projection (Figure 10), these results consistently indicate that the proposed model learns meaningful and well-structured representations of student behavior, with generally good separability between classes.

The projection shows a generally well-separated structure between at-risk and successful students, with some overlap, indicating that the learned representations capture meaningful linear differences in student behavior.

The visualization reveals compact clusters with generally well-separated structure and limited overlap, preserving both local and global structure in the learned feature space.

**Figure 13:**
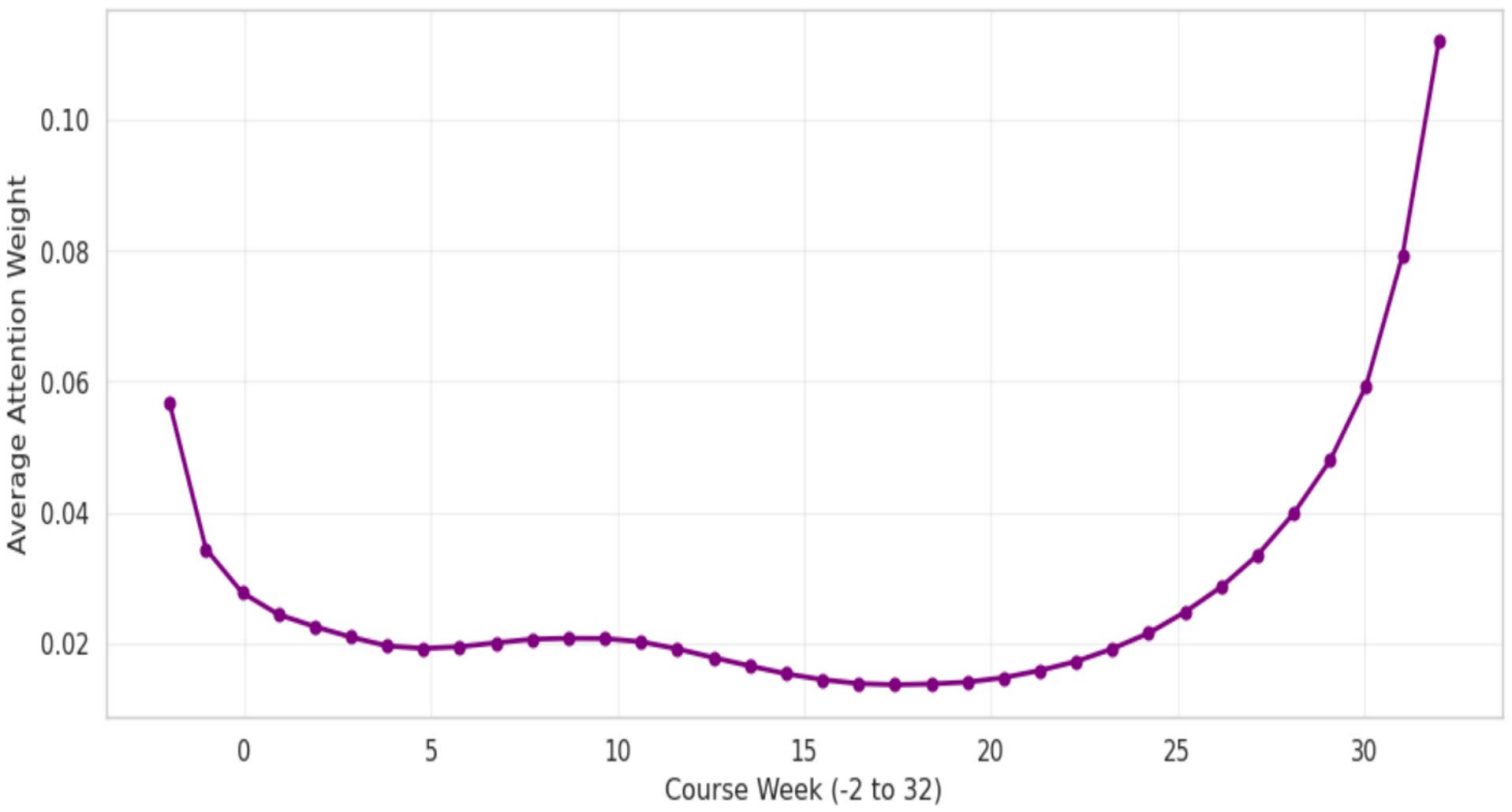
Temporal feature importance (attention mechanism analysis).

Although some overlap between the red and blue clusters is observed in localized regions of the t-SNE projection, the confusion matrix indicates that 141 instances correspond to students who exhibited behavioral patterns associated with passing but ultimately failed academically. Despite these overlapping cases, the t-SNE visualization supports the conclusion that the combination of static and temporal features captures meaningful differences between student outcome groups. Overall, the learned latent representations provide a discriminative structure that reflects underlying variations in learning behavior and performance. The attention mechanism offers additional insight into how the temporal component of the model contributes to prediction. By assigning different importance weights to each week of activity, the model highlights periods that are more influential for determining final outcomes. The distribution of attention weights exhibits a U-shaped pattern across the course timeline. In particular, the weeks preceding registration and the initial weeks of the course (up to Week 0) receive higher weights, indicating that early engagement behaviors are strongly associated with subsequent academic performance. This pattern is illustrated in Figure 13 and suggests that early interactions with course materials and timely registration are informative indicators of later success.

Following the initial phase of the course, engagement patterns remain relatively stable during the middle of the semester and contribute less to outcome differentiation. In contrast, a marked increase in attention weights is observed toward the end of the term (Weeks 25–32), with values rising sharply and exceeding 0.10. This trend indicates that student activity during the final phase of the course plays a particularly important role in determining academic outcomes. The observed pattern suggests that interactions with learning materials shortly before final assessments are strongly associated with subsequent performance. From an instructional perspective, this finding is consistent with the view that concentrated effort and increased participation toward the end of a course are informative indicators of whether students are likely to succeed or encounter difficulties. These late-stage behaviors may therefore serve as useful signals for targeted academic support. Overall, the results demonstrate that the proposed hybrid model with an attention mechanism outperforms models based solely on demographic features or basic behavioral representations, achieving a ROC-AUC of 0.95 and a weighted F1-score of 0.90. In addition to improved predictive performance, the model provides interpretable insights into temporal patterns of learning behavior. The attention analysis highlights the late phase of the semester as particularly influential for outcome prediction, supporting the effectiveness of the proposed approach for early risk identification and timely academic intervention in virtual learning environments.

## 4 Discussion

To situate the proposed approach within the existing literature, we compare it with representative studies that have evaluated predictive models on the OULAD benchmark. Table 6 provides a qualitative comparison of modeling paradigms, temporal modeling capabilities, and interpretability. In addition, Table 7 presents a quantitative comparison with prior OULAD-based models.

**Table 6:** Qualitative comparison of representative OULAD-based studies.

Study	Dataset	Model family	Temporal modeling	Interpretability	Reported metrics (as stated)
Althibyani (2024)	OULAD	Classical ML	mostly aggregated	limited	reports ML comparisons
Shayegan and Akhtari (2024)	OULAD	Stacking/ensemble	aggregated	limited	stacking results
Torkhani and Rezgui (2025)	OULAD	DL (incl. LSTM)	+	not focus	LSTM accuracy ~83.41%
Kalita et al. (2025)	OULAD	LSTM + SHAP	+	+	accuracy/F1 reported
Jha et al. (2019)	OULAD	Attention LSTM	+	+	attention-based LSTM described
Our study	OULAD	Bi-LSTM + MLP + attention	+	+	ROC-AUC 0.95, F1 0.90

**Table 7:** Quantitative comparison with prior OULAD-based models.

Model	Source	Metric	Reported value
Classical ML models	Althibyani (2024)	Accuracy	0.87–0.88
Stacking ensemble	Shayegan and Akhtari (2024)	Accuracy	0.88–0.89
LSTM	Torkhani and Rezgui (2025)	Accuracy	0.834
Attention-based DL	Jha et al. (2019)	Accuracy	0.84–0.86
LSTM + SHAP	Kalita et al. (2025)	Accuracy / F1	0.88–0.90
Proposed hybrid model	This study	ROC-AUC	0.9497
		Weighted F1	0.9008

Table 7 provides a quantitative comparison with prior OULAD-based models to complement the qualitative analysis presented in Table 6.

It should be noted that the compared models were not re-implemented under a unified experimental setup, and reported results are taken from the respective original studies. Therefore, the comparison is indicative rather than strictly controlled.

The results indicate that integrating temporal behavioral sequences with static student characteristics provides a useful representation for modeling student outcomes in online learning environments. The observed performance differences between hybrid and single-modality models suggest that temporal dynamics of engagement play a meaningful role in distinguishing students who complete a course from those who withdraw. A central component of the proposed framework is the temporal attention mechanism, which allows the model to assign different weights to different stages of the learning process. The attention patterns indicate that early course activity and engagement toward the end of the course contribute more to outcome prediction than activity in the middle phase. This suggests that changes in engagement at specific stages of a course may be more closely related to subsequent academic outcomes. Sequential models that treat all time steps as equally informative may therefore overlook periods that are more indicative of risk. The comparison between models relying only on demographic information and those incorporating behavioral data highlights the limited capacity of static features to reflect short-term changes in learner engagement. Behavioral indicators derived from platform interactions provide more direct signals of how students participate in the learning process over time. Combining these sources of information supports a more nuanced representation of learning trajectories. From a practical perspective, the attention mechanism contributes to the interpretability of the model by indicating which phases of a course are more informative for prediction. This may support instructors and academic advisors in monitoring shifts in student participation patterns. Although the analysis is based on multiple modules and course presentations from the OULAD dataset, further evaluation on other platforms is required to examine the extent to which these patterns generalize across contexts. Finally, the use of cost-sensitive learning reflects the practical priorities of early warning systems, where failing to identify a student in need of support may have more serious implications than generating a limited number of false alerts. Maintaining adequate recall for the at-risk group is therefore relevant for potential applications in educational settings.

As demonstrated in Table 6, the model retains strong predictive capability even when restricted to early and mid-course data, confirming its suitability for early warning scenarios.

While the individual components of the proposed architecture, such as dual-stream networks, attention mechanisms, and cost-sensitive learning, have been explored in prior research, the contribution of this work lies in their integration within a unified framework tailored to modeling multimodal learning behavior. In particular, the proposed approach combines temporal engagement sequences with static student characteristics and incorporates attention-based analysis to examine the relative importance of different learning phases. This enables not only improved predictive performance but also provides interpretable insights into how student behavior evolves over time.

## 5 Conclusion

This study examined a hybrid deep learning framework for identifying students at risk in online learning environments by combining temporal behavioral patterns with static student characteristics. The evaluation on the OULAD dataset indicates that this hybrid configuration provides a basis for modeling student outcomes in settings where learning behavior evolves over time. The analysis of temporal attention weights suggests that different phases of a course contribute unevenly to outcome prediction, with early engagement and activity close to assessment deadlines being more informative than the middle phase. This observation points to the relevance of considering temporal heterogeneity in student behavior when designing predictive models for learning analytics. Several limitations should be acknowledged. Clickstream data describe the quantity of interaction but do not capture qualitative aspects of cognitive engagement. In addition, the most informative predictive signals tend to appear in later stages of a course, which constrains the window for early pedagogical intervention. The model was evaluated on a single benchmark dataset, and further validation across different institutional and cultural contexts is needed. Future work will focus on incorporating textual data from discussion forums and written assignments through natural language processing methods, conducting systematic ablation studies to assess the contribution of individual model components, and exploring earlier indicators of disengagement. Overall, the proposed approach provides a foundation for the development of interpretable, data-driven tools to support student monitoring and targeted academic support in online learning environments.

## Data Availability

The datasets analyzed in this study are publicly available. The Open University Learning Analytics Dataset (OULAD) can be accessed at: https://analyse.kmi.open.ac.uk/open-dataset.

## References

[ref1] Alghamdi S. Soh B. Li A. (2025). A comprehensive review of dropout prediction methods based on multivariate analyzed features of MOOC platforms. Multim. Technol. Interact. 9:3. doi: 10.3390/mti9010003

[ref2] Althibyani A. (2024). Predicting student success in MOOCs: a comprehensive analysis using machine learning models. PeerJ Computer Sci. 10:e2221. doi: 10.7717/peerj-cs.2221, 39678289 PMC11639146

[ref3] Baranyi M. Moontay R. (2020). “Interpretable deep learning for university dropout prediction,” in Proceedings of the 21st Annual Conference on Information Technology Education, (), 13–19.

[ref4] Basnet R. B. Johnson C. Doleck T. (2022). Dropout prediction in MOOCs using deep learning and machine learning. Educ. Inf. Technol. 27, 11499–11513. doi: 10.1007/s10639-022-11068-7

[ref5] Betaitia Z. Chefrour A. Drissi S. (2025). Exploring dropout rates in MOOC research: a bibliometric analysis. J. Learn. Dev. 12, 76–92. doi: 10.56059/jl4d.v12i1.1597

[ref6] Cai L. Zhang G. (2021). “Prediction of MOOCs dropout based on WCLSRT model,” in In 2021 IEEE 5th International Conference on Advanced Information Technology, Electronic and Automation Control (IAEAC), (IEEE), 780–784.

[ref7] Chen M. Wu L. A. (2021). A dropout prediction method based on time-series models in MOOCs. J. Phys. Conf. Ser. 1774:012065. doi: 10.1088/1742-6596/1774/1/012065

[ref8] Dai Z. Fu J. Zhu Q. Cui H. Li X. Qi Y. (2020). Local contextual attention with hierarchical structure for dialogue act recognition. arXiv. doi: 10.48550/arXiv.2003.06044

[ref9] Devi P. Rani R. Kumar A. Siwach A. K. (2025). Mapping the landscape of MOOCs research: a bibliometric analysis of the top 100 cited papers. DESIDOC J. Lib. Inform. Technol. 45, 458–467. doi: 10.14429/djlit.20188

[ref10] Jha N. I. Ghergulescu I. Moldovan A. N. (2019). OULAD MOOC dropout and result prediction using ensemble, deep learning and regression techniques. In CSEDU (pp. 154–164).

[ref11] Kalita E. El Aouifi H. Kukkar A. Hussain S. Ali T. Gaftandzhieva S. (2025). LSTM-SHAP based academic performance prediction for disabled learners in virtual learning environments: a statistical analysis approach. Soc. Netw. Anal. Min. 15, 1–23. doi: 10.1007/s13278-025-01484-1

[ref12] Liu H. Chen X. Zhao F. (2024). Learning behavior feature-fused deep learning network model for MOOC dropout prediction. Educ. Inf. Technol. 29, 3257–3278. doi: 10.1007/s10639-023-11960-w

[ref13] Long F. Zhou K. Ou W. (2019). Sentiment analysis of text based on bidirectional LSTM with multi-head attention. IEEE Access 7, 141960–141969. doi: 10.1109/ACCESS.2019.2942614

[ref14] Mubarak A. A. Cao H. Zhang W. (2021). Visual analytics of video-clickstream data and prediction of learners’ performance using deep learning models in MOOCs. Comput. Appl. Eng. Educ. 29, 710–732. doi: 10.1002/cae.22328

[ref15] Open University Learning Analytics Dataset (OULAD). Available online at: https://analyse.kmi.open.ac.uk/open-dataset (Accessed 15 November 2025).

[ref16] Pulikottil S. C. Gupta M. (2020). ONet: A temporal meta-Embedding Network for MOOC Dropout Prediction. In Proceedings of the 2020 IEEE International Conference on Big Data (pp. 5209–5217).

[ref17] Shayegan M. J. Akhtari R. (2024). A stacking machine learning model for student performance prediction based on class activities in E-learning. Comput. Syst. Sci. Eng. 48, 1251–1272. doi: 10.32604/csse.2024.052587

[ref18] Torkhani W. Rezgui K. (2025). OULAD MOOC Student Performance Prediction using Machine and Deep Learning. In Proceedings of the International Conference on Decision Aid and Artificial Intelligence (ICODAI 2024) (12: 228). Springer Nature.

[ref19] Zheng Y. Gao Z. Wang Y. (2020). MOOC dropout prediction using FWTS-CNN model based on fused feature weighting and time series. IEEE Acc. 8, 225324–225335. doi: 10.1109/ACCESS.2020.3045157

